# Caregiver-Centered Care Health Workforce Competencies: Developing Consistent Support for Family Caregivers

**DOI:** 10.1093/geroni/igaa057.050

**Published:** 2020-12-16

**Authors:** Jasneet Parmar, Sharon Anderson, Lisa Poole, Wendy Duggleby, Jayna Holyroyd-Leduc, Suzette Brémault-Phillips, Pollard Cheryl, Anwar Haq

**Affiliations:** 1 University of Alberta, Edmonton, Alberta, Canada; 2 Dementia Connections Magazine, Calgary, Alberta, Canada; 3 University of Calgary, Calgary, Alberta, Canada; 4 McEwan University, Edmonton, Alberta, Canada; 5 Covenant Health Network of Excellence in Seniors' Health and Wellness, Edmonton, Alberta, Canada

## Abstract

Family caregivers [FCGs] are the backbone of the health system. They provide over 80% of the care for people with dementia, chronic illnesses and impairments. Despite evidence of their contributions and consequences of caregiving, support for FCGs has not been a health system priority. Education to prepare health providers to effectively identify, engage, assess, and support FCGs throughout the care trajectory is an innovative approach in addressing inconsistent system of supports for FCGs. We report on development and validation of the Caregiver-Centered Care Competency Framework to help with curricular design and subsequent evaluation of effectiveness of care providers working within healthcare settings to engage and support FCGs. We used a three round modified Delphi approach. An expert panel of 42 international, national, and provincial stakeholders agreed to participate. In the first 2 rounds, multi-level, interdisciplinary participants, rated the indicators in terms of importance and relevance. In the 3rd round consensus meeting, participants validated the six competency domains, including indicators in small group sessions. Thirty-four experts (81%) participated in the round 1, 36 (85.7%) in round 2, and 42 people (100%) in round 3. There was stable consensus across all three rounds, 96.07% of participants rated the indicators as essential or important (Round 1, 95.81%; Round 2, 94.15; Round 3, 98.23%). FCG research has been primarily focussed on educating FCGs to provide care. These competencies will shape the design of educational curricula and interdisciplinary training programs aimed at supporting the health and social care workforce to provide caregiver-centered care.

